# Supplementary material for: Birnaviridae Virus Factories Show Features of Liquid-Liquid Phase Separation and Are Distinct from Paracrystalline Arrays of Virions Observed by Electron Microscopy
Source: J Virol. 2022 Mar 23;96(6):e02024-21. doi: 10.1128/jvi.02024-21 (PMC8941928; doi:10.1128/jvi.02024-21)
Supplement: Supplemental file 4 — Supplemental Movie Legends . Download jvi.02024-21-s0004.pdf, PDF file, 0.2 MB [file jvi.02024-21-s0004.pdf]

Movie S1. IBDV virus factories were present in infected, mock-treated cells imaged live. DF-1 cells were transfected with GFP1-10 and infected with the PBG98-VP1-GFP11 virus. Infected cells were mock-treated with media 16hpi and imaged live over a time course of 17 minutes. Fifty z-stacks were imaged in total, with each stack being acquired at an interval of 20 seconds.

Movie S2. IBDV virus factories dissolved in infected cells treated with 4% 1,6-hexanediol and imaged live. DF-1 cells were transfected with GFP1-10 and infected with the PBG98-VP1-GFP11 virus. Infected cells were treated with a solution of 4% 1,6-hexanediol 16hpi and imaged live over a time course of 17 minutes. Fifty z-stacks were imaged in total, with each stack being acquired at an interval of 20 seconds.

Movie S3. Paracrystalline arrays of virions detected by electron tomography. DF-1 cells were infected with IBDV strain PBG98 and prepared for TEM 18hpi. Serial electron microscopy datasets were collected every 1º over a 120º tilt series and reconstructed. 10nm gold antibodies acted as fiducial markers.
